# Mediating mechanisms in the discrimination – Mental health link among Mexican-origin adolescents: A latent growth curve mediation analysis

**DOI:** 10.1017/S0954579426101163

**Published:** 2026-02-03

**Authors:** Irene J.K. Park, Lijuan Wang, Yuan Fang, Kristin Valentino, Tiffany Yip, Jenny Zhen-Duan, Mario Cruz-Gonzalez, Kiara Alvarez, Margarita Alegría

**Affiliations:** 1 Department of Psychiatry, Indiana University School of Medicine-South Bend, South Bend, IN, USA; 2 Department of Public Health & Epidemiology, College of Medicine and Health Sciences, Khalifa University of Science and Technology, Abu Dhabi, United Arab Emirates; 3 Department of Psychology, University of Notre Dame, Notre Dame, IN, USA; 4 Department of Psychology, Fordham University, New York, NY, USA; 5 Disparities Research Unit, Department of Medicine, Massachusetts General Hospital, Boston, MA, USA; 6 Department of Psychiatry, Harvard Medical School, Boston, MA, USA; 7 Department of Medicine, Harvard Medical School, Boston, MA, USA; 8 Department of Health, Behavior and Society, Johns Hopkins Bloomberg School of Public Health, Baltimore, MD, USA

**Keywords:** Anger, Mexican-origin adolescents, racial/ethnic discrimination, racism-related vigilance, vicarious racism

## Abstract

The present three-wave longitudinal study tested two transdiagnostic mediators – anger and racism-related vigilance – of the link between racism and internalizing and externalizing problems. At Wave 1, the sample included 344 Mexican-origin adolescents (*M_age_* = 13.5 years; 51.7% male, 45.9% female; 2.3% non-binary) residing in the Midwestern United States. Data across the three waves were collected from April 2021 through October 2024. The study examined how both direct and vicarious racism were related to internalizing and externalizing problems over time. Results from latent growth curve mediation analyses indicated that outward anger expression was a significant mediator; both direct and vicarious racism at Wave 1 were significantly associated with higher levels of anger at Wave 2, which in turn, were associated with higher levels of internalizing and externalizing problems at Wave 3. Racism-related vigilance was a significant mediator of the association between vicarious racism and internalizing problems only, according to results from post hoc sensitivity analyses. Implications for future theory, research, and clinical practice are discussed to help mitigate the effects of racism in new migration contexts for this vulnerable population.

Racism has been declared “a public health problem, threat, and crisis” in the United States (Hall & Boulware, 2023). According to James Jones (1972), racism is defined as “the exercise of power against a racial group defined as inferior by individuals and institutions with the intentional or unintentional support of the entire culture” (p. 117) and manifests at the institutional, cultural, and individual levels. As a driver of health inequities among racially and ethnically minoritized populations, racism is linked with adverse effects on physical and mental health (Paradies et al., 2015; Williams & Mohammed, 2009).

Racial/ethnic discrimination (racism experienced at the individual level, Priest et al., 2017) may be defined as “unfair treatment due to an individual’s race or ethnicity” (Contrada et al., 2000). Racial/ethnic discrimination has been consistently associated with poor mental health outcomes not only in adults (Paradies et al., 2015) but also among racially/ethnically minoritized children and adolescents (Benner et al., 2018; Priest et al., 2013). In the United States, Latinx adolescents represent the largest racial/ethnic minority population of youth (Jones et al., 2021), and Mexican-origin youth comprise the largest group therein (Pew Research Center, 2022). As Mexican-origin adolescents experience high rates of racial/ethnic discrimination (Zeiders et al., 2021), there are adverse consequences on their mental health (Benner et al., 2018). Latinx youth have reported higher levels of internalizing and externalizing problems compared to other racial/ethnic groups (McLaughlin et al., 2007). One prospective study found that racial/ethnic discrimination was associated with higher levels of internalizing and externalizing problems among Mexican-origin youths over time (Berkel et al., 2010).

To better understand how racism is related to youth mental health, it is vital to recognize the detrimental effects of not only *direct* but also *indirect* or *vicarious* forms of racism (Heard-Garris et al., 2018). Vicarious racism has been defined as “secondhand exposure to the racial discrimination and/or prejudice directed at another individual” (Heard-Garris et al., 2018, p. 235). In the present study, vicarious racism is defined as hearing about or seeing other Latinx individuals being treated unfairly because of their race or ethnicity. Examples of vicarious racism include hearing or seeing racist things about Latinx people posted on social media or hearing public figures (e.g., entertainers, politicians) saying racist things about Latinx people. In a highly charged and polarized sociopolitical climate, negative media representations of racially and ethnically minoritized populations experienced as vicarious racism can be an especially salient threat to adolescent well-being (Cohen et al., 2021; Heard-Garris et al., 2021). Given the critical developmental changes in adolescence and the importance of navigating identity, experiences of both direct and vicarious racism can represent assaults on youths’ racial and ethnic identity and collective group esteem.

To effectively intervene in or prevent mental health problems for this significant population, it is imperative to identify theory-driven mediators in the link between discrimination and mental health, especially among Mexican-origin youth. By studying mediators, we can identify how discrimination, in its various forms, is harmful to the mental health of Latinx adolescents. Pinpointing these mechanisms can aid the design of developmentally and culturally appropriate intervention and prevention programs and, ultimately, reduce the potential threat posed by racism to this population.

## Theoretical foundations

A socioecological approach (García Coll et al., 1996; Suárez-Orozco et al., 2018) informed the present study to understand the well-being of minoritized and immigrant youth given their social position and experiences of racism, which operate through multiple environmental contexts, including political and social contexts of reception. The integration of theoretical frameworks developed by Harrell (2000) and Brondolo et al. (2018) informed our hypothesized model whereby anger and racism-related vigilance, respectively, mediate associations between youth experiences of discrimination (direct and vicarious) and their mental health.

According to Harrell’s “Model of Racism-Related Stress and Well-Being” (2000), interpersonal racism can be expressed through both direct and vicarious experiences. Thus, the present study operationalizes racism in a two-pronged manner by assessing adolescents’ direct and vicarious experiences of racism and discrimination. Moreover, Harrell (2000) identified several “affective and behavioral responses to racism-related stressors” (p. 49) as intrapersonal mediators, including anger; this informed the present study’s focus on anger as a potential mediator. Harrell theorized that emotional responses like anger mediate the effects of racism on a variety of mental health and physical outcomes. The present study outcomes (internalizing and externalizing problems) likewise reflect the reality that racism can impact well-being in a wide-ranging manner.

Brondolo et al.’s (2018) “Biopsychosocial Model of the Role of Social Cognitive Processes in the Relationship of Discrimination to Health” emphasizes the mediating role of social cognition in the discrimination – health link. Brondolo et al. (2018) contend that racial/ethnic discrimination elicits negative relational schemas and threat appraisals, such as racism-related vigilance, which in turn lead to negative mood and emotional reactivity as well as risky or unhealthy externalizing behaviors. Guided by this framework, the present study focused on racism-related vigilance as a potential mediator in the discrimination – mental health link. Given that Brondolo et al.’s (2018) framework, like Harrell’s (2000) model, posits that exposure to discrimination increases the risk for a range of mental health problems over time, the present study focused on both internalizing and externalizing problems.

Taken together, these multidimensional approaches elucidate important underlying mechanisms that can explain how racism is associated with poor mental health outcomes. By examining these mechanisms over time, we can better understand how initial levels of, and changes in, these constructs are associated with initial levels of, and changes in, psychopathology during the critical developmental period of adolescence for Mexican-origin youth. This nuanced understanding of mechanisms can be used to titrate prevention and intervention efforts to be more effective and culturally responsive for this population.

## Empirical evidence for mediating mechanisms

Anger and racism-related vigilance were selected as hypothesized mediators in the association between racism and mental health not only for theoretical reasons but also based on the extant empirical literature. From a developmental psychopathology perspective, anger expression (as a form of emotion dysregulation) and vigilance represent important transdiagnostic mechanisms that can compromise mental health outcomes from adolescence through adulthood. Emotion dysregulation and vigilance, respectively, are key features observed in various forms of psychopathology such as internalizing and externalizing disorders (Beauchaine & Cicchetti, 2019; Woody et al., 2022). In line with the principle of multifinality (Cicchetti & Rogosch, 1996), exposure to racial/ethnic discrimination is associated with a wide range of negative psychological and behavioral outcomes (e.g., Paradies et al., 2015), such as externalizing and internalizing problems, which are typically precursors to more severe forms of psychopathology (van der Ende et al., 2020).

Two recent systematic reviews have identified emotion dysregulation and vigilance as key transdiagnostic mediators with explanatory power in the link between racism-related stressors and adolescent mental health (Roach et al., 2023; Woody et al., 2022). We acknowledge that both anger and racism-related vigilance may represent reasonable responses to direct and vicarious racism. Yet, cumulatively over time and at high intensity, both could have adverse effects on emotional and physical health. Given the enormous social, emotional, biological, and physiological changes occurring during adolescence, it is especially valuable to conduct analyses and test models that incorporate change over time. However, there is a paucity of literature that focuses on Latinx adolescents and captures change longitudinally.

### Anger

The association between emotion dysregulation (including anger dysregulation) and the development of psychopathology has been well established (Beauchaine & Cicchetti, 2019; Kerr & Schneider, 2008). Given the acquisition of increasingly complex emotion regulation skills during adolescence (Dahl & Spear, 2004; Ladouceur et al., 2012), emotion regulation is particularly salient during this developmental stage. Less research has investigated the link between direct or vicarious racism and emotion regulation (and specifically anger expression), particularly at micro- and macro-level time scales (e.g., via daily diary or longitudinal studies). Some recent exceptions are noteworthy (Park et al., 2024; Roach et al., 2023). For example, Roach et al. (2023) concluded from their systematic review that anger was the emotion dysregulation construct most frequently studied and associated with race-related stress and trauma. These empirical findings guided the present study’s focus on anger expression as a hypothesized mediator.

One three-wave study was the first to provide longitudinal evidence that outward anger expression mediates the link between racial/ethnic discrimination and anxiety and depression among Mexican American adolescents (Park et al., 2017). In the face of unjust treatment as a function of one’s ethnicity or race, anger is a powerful emotion that is often evoked. Yet, these findings indicated that anger expression has negative consequences for adolescent mental health, namely, higher levels of anxiety and depressive symptoms. This prior study also tested two other forms of anger regulation (anger suppression and anger control), but results indicated that they did not significantly mediate the association between discrimination and either anxiety or depressive symptoms. Therefore, in the present study, we chose to test outward anger expression as one potential mediator of the racism – mental health link. In the prior study, however, time intervals were spaced 4–6 months apart, and it is not known whether these mediation effects would hold over more extended periods of adolescent development, as in the present research.

Another recent study (from the same larger parent project as the current study; Park et al., 2024) was the first daily diary study to show that outward anger expression mediated the association between racial/ethnic discrimination and negative affect and stress at a micro-time level, examining both within-person and between-person mediation effects on a daily basis. Taken together, these two studies provide evidence that anger is a critical mediator both at the daily level and at a longer-term level (over 4–6 months) in explaining why exposure to racial/ethnic discrimination is associated with distress and symptoms of anxiety and depression. The present study extends this work to the macro-time level through a longitudinal investigation with yearly assessments over three years, by examining both internalizing and externalizing problems, and by examining both direct and vicarious forms of racism and discrimination.

### Racism-related vigilance

A form of cognitive appraisal, racism-related vigilance is a state of psychological arousal to cope with potential discrimination experiences (Williams, 2018) and can serve as an adaptive mechanism to ward off such threats. However, when vigilance becomes a long-term and chronic form of coping, it can have adverse effects on mental and physical health (Himmelstein et al., 2015; LaVeist et al., 2014; Pichardo et al., 2021; Woody et al., 2022).

Particularly for marginalized Latinx youth in unreceptive environments (Espinola et al., 2019) or new immigrant destinations such as the Midwestern United States, racism can trigger racism-related vigilance and, in turn, adversely affect mental health (e.g., Park et al., 2024). Recent research highlights vigilance as a critical yet understudied transdiagnostic mechanism that may help explain mental and physical health disparities in racially minoritized groups (Hicken et al., 2014; Woody et al., 2022).

Several recent studies have examined the mediating role of racism-related vigilance in the association between racism-related stressors and mental health (Himmelstein et al., 2015; Mikrut et al., 2022; Pichardo et al., 2021). One study of racially diverse (23.8% Latinx) adults found that social vigilance was *not* a significant mediator of the link between discrimination and depressive symptoms (Mikrut et al., 2022). In contrast, Pichardo et al. (2021) found that racism-related vigilance was a significant mediator of the association between racial/ethnic discrimination and depressive symptoms in a sample of Latinx college students. Whereas these studies are useful for theory-building, they were cross-sectional with the attendant design limitations and focused on adults or college students. Cross-sectional studies cannot account for temporal ordering in mediation mechanisms and can yield misleading inferences for both direct and indirect effects (e.g., Maxwell and Cole, 2007). The present study addressed both the design and sample limitations of prior research by employing a longitudinal design and sampling younger adolescents who may be more vulnerable to identity threats via racism in a period of developmental changes.

One recent daily diary study provided evidence at the daily level that vigilance mediates the association between racial/ethnic discrimination and distress in a sample of Mexican-origin youth (Park et al., 2024). This was an important contribution to the literature because it was the first empirical test of vigilance as a mediating mechanism at the daily level in a minoritized population of color. The results showed that exposure to racial/ethnic discrimination among Mexican-origin adolescents is associated with more vigilance (e.g., more threat appraisals, more monitoring of their environment) which in turn, takes a toll on well-being even in the short term with regard to negative affect and stress. To our understanding, no research to date has yet captured developmental changes in a mediation model testing racism-related vigilance as a proposed mediator in the association between discrimination and internalizing and externalizing problems in any sample of minoritized adolescents, including Latinx or Mexican-origin adolescents.

## The current study

The current study is poised to contribute to the extant literature by integrating multidisciplinary theoretical models (Brondolo et al., 2018; García Coll et al., 1996; Harrell, 2000; Suárez-Orozco et al., 2018), building upon prior empirical work (e.g., Park et al., 2017, 2024; Roach et al., 2023; Woody et al., 2022), and capitalizing on the advantages of a multi-level, longitudinal design. First, the present study tests two transdiagnostic mediating mechanisms, namely anger (Harrell, 2000) and racism-related vigilance (Brondolo et al., 2018), theorized to help explain the link between racial/ethnic discrimination and mental health. Second, the present study not only examines direct encounters with racial/ethnic discrimination but also vicarious experiences of racism (Harrell, 2000). Third, we test these putative mechanisms in a sample of Mexican-origin adolescents living in a relatively novel immigrant destination. Because much of the scientific knowledge base on Latinx youth is based on samples derived from large metropolitan areas with substantial Latinx populations (such as Miami, New York City, Los Angeles, or the Southwestern US), it is especially important to sample Latinx adolescents in a new migration context to obtain a comprehensive estimate of the detrimental effects of racism on mental health. Next, the three-wave (yearly) longitudinal design affords the opportunity to assess changes over time and test mediation mechanisms using growth curve mediation analyses. We investigate these longitudinal processes during the developmentally critical transition into adolescence. How adolescents express or handle their anger and the degree to which they develop racism-related vigilance in response to direct and vicarious racism are especially salient at this developmental stage, with potential long-term repercussions for adulthood.

Thus, we tested the following hypotheses:

(H1) The association between racism and adolescent mental health will be mediated by anger and racism-related vigilance. Initial levels of direct or vicarious experiences of racism at Wave 1 will be associated with anger and vigilance, respectively, at Wave 2, which, in turn, will be associated with final levels of internalizing and externalizing symptoms at Wave 3.

(H2) Changes in direct or vicarious experiences of racism over time (between Waves 1 and 3) will be associated with changes in anger and vigilance over time (between Waves 1 and 3), which, in turn, will be associated with changes in internalizing and externalizing symptoms over time (between Waves 1 and 3).

Although the detrimental effects of both direct and vicarious racism have been well established (Benner et al., 2018; Heard-Garris et al., 2018; Priest et al., 2013), it is still unclear the degree to which they may be similar or distinct from one another vis-à-vis their effects on these hypothesized mediators and adolescent mental health. Therefore, we do not advance specific hypotheses about direct versus vicarious racism; thus, in the growth curve mediation models, we test each predictor, mediator, and outcome variable separately to allow for a detailed examination of each.

## Method

### Participants

Adolescent participants were drawn from the *Seguimos Avanzando* study, a three-wave, multi-level longitudinal study of racism and discrimination-related stressors and mental health among Mexican-origin adolescents and their primary caregivers in Northern Indiana (Alegría et al., 2024). The intervals between consecutive waves were spaced 9 to 15 months apart. At Wave 1 (W1), participants were 344 Mexican-origin adolescents (12–16 years old; *M*_age_ = 13.47; *SD* = 1.08) living in the Midwestern United States. The W1 adolescent sample was 45.93% female (*n* = 158), 51.74% male (*n* = 178), and 2.33% (*n* = 8) non-binary/third gender. At Wave 2 (W2) and Wave 3 (W3), the data analytic samples comprised *n* = 267 and *n* = 261 adolescents, respectively. We focused on this age range to observe developmental changes associated with the transition into adolescence.

Most W1 adolescents were born in the U.S. (93.02%, *n* = 320). In contrast, most parents (92.54% of mothers and 94.89% of fathers) were born in Mexico, indicating that this sample is predominantly comprised of immigrant families. The majority of the W1 adolescents (80.52%, *n* = 277) reported living in two-parent families, with 13.37% (*n* = 46) from single-mother families and 5.81% (*n* = 20) reporting some “Other” family structure (one participant missing data). The median annual family income for mothers was $20,000–29,000 and $40,000–49,000 for fathers. Parents’ median and modal education level was “9th grade” for both mothers (16.56%) and fathers (19.75%).

### Recruitment and procedures

Mexican-origin adolescents and their primary caregivers were recruited through local churches, public schools, and community-based organizations in Northern Indiana from April 2021 through December 2022, employing a purposive sampling strategy. This region represents a relatively new migration area for Latinx families. The present study utilized an ethnic-homogenous design (Roosa et al., 2008) and best practice recruitment procedures recommended for recruiting Latinx families (Martinez et al., 2012).

To participate in the current study, families were required to meet specific eligibility criteria. Inclusion criteria included: (a) the family has an adolescent 12–15 years old, of Mexican descent, (b) both of the adolescent’s biological parents are of Mexican origin, and (c) the adolescent is residing with either a biological parent or a legal guardian, also of Mexican origin. Exclusion criteria included: (a) the adolescent has a severe learning or developmental disability that would hinder survey completion, and (b) the family participated in our prior research which assessed similar constructs (e.g., Park et al., 2017). Trained bilingual staff obtained informed consent from parents and assent from adolescent participants in their preferred language (English or Spanish) before administering the survey to adolescents either in-person (74.7%) or virtually via Zoom (25.3%). Adolescent surveys were electronic and self-administered on devices (e.g., tablets or laptops).

All participating family members were compensated for their time through gift cards. Participating adolescents received a $30 gift card at W1, $30 at W2, and $40 at W3. Each parent received a $35 gift card at W1, $35 at W2, and $50 at W3. The IRB at the University of Notre Dame approved this study with ceded reviews from other participating institutions. For more details on all study procedures, please see Alegría et al. (2024).

### Measures

All measures in the present study were available to youths in both English and Spanish; most youths (96.2%) opted for the English language format.

#### Demographic background

Adolescent participants provided self-reports of their age, gender, ethnicity, length of United States residency, birthplace, and family structure; mothers and fathers provided self-reports of their family’s annual household income and education levels.

#### Racial/ethnic discrimination

Adolescents’ lifetime experience of direct racial/ethnic discrimination was assessed using an adapted 8-item version of the Perceptions of Racism in Children and Youth (PRaCY; short version; Pachter et al., 2010). Adolescents were asked about discrimination due to the color of their skin, language, or accent, or because of their culture or country of origin, using a dichotomous response format (“Yes” = 1; “No” = 0). Sample items include: “Have you ever had someone make a bad or insulting remark about your race, ethnicity, or language?” and “Have you ever seen your parents or other family members treated unfairly or badly because of the color of their skin, language, accent, or because they came from a different country or culture?” A sum score was created, with higher scores indicating more exposure to racial/ethnic discrimination (range: 0–8). The internal consistency of the 8-item PRaCY was adequate across the three waves of the present study, with Cronbach’s alphas of .74 (W1), .79 (W2), and .79 (W3).

#### Vicarious experiences of racism

Adolescents’ vicarious experiences of discrimination were assessed using the 8-item Vicarious Experiences of Racism Scale (VERS; Chae et al., 2021; Yip et al., 2024). Five items asked adolescents how frequently they saw or heard about other Latinx individuals experiencing racism on a 7-point scale (0 = *Never*; 6 = *Almost every day*). Sample items included: “How often did you hear about or see other Latinos in public being treated unfairly because of their race?” and “How often did you hear about or see racist things about Latinos posted on social media?” The mean of these five frequency items was computed to create a frequency score. Internal consistency was adequate, with Cronbach’s alphas of .83 (W1), .86 (W2), and .85 (W3).

Three items assessed the impact on adolescents, in terms of how much they “thought about,” were “distressed or bothered,” and were “concerned or worried” about these experiences of vicarious racism, with responses recorded on a 5-point scale (0 = *Never*; 4 = *Always*). Cronbach’s alphas of the impact items were .79 (W1), .79 (W2), and .85 (W3). The VERS was scored using a weighted average, calculated by multiplying the overall frequency score by the mean of the three “impact” items (Chae et al., 2021). Higher VERS scores indicated higher levels of adolescents’ vicarious experiences of racism. The “impact” items were similar to core items on the YSR internalizing problems scale. To remove this potential overlap, unweighted averages (without using the “impact” items) were used in a post hoc sensitivity analysis.

#### Anger

Adolescents’ outward anger expression was assessed using the 5-item *Anger Expression-Out* subscale of the State-Trait Anger Expression Inventory-2 Child/Adolescent version (STAXI-2 C/A; Brunner & Spielberger, 2009). Youths were asked how often they express anger toward objects or people in their environment using a 3-point response format (1 = *Hardly ever;* 3 = *Often*). A mean score was calculated, with higher scores indicating higher levels of outward anger expression. Internal consistency was adequate across the three waves of the present study, with Cronbach’s alphas of .73 (W1), .74 (W2), and .68 (W3).

#### Racism-related vigilance

Adolescents’ racism-related vigilance was assessed using the 6-item Heightened Vigilance Scale (HVS; Clark et al., 2006; Hicken et al., 2014). Sample items include: “How often do you try to prepare for possible insults before leaving home?” and “How often do you try to avoid certain social situations and places?” Participants rated each item using a 5-point response format (1 = *Very often*; 5 = *Never*). All items were reverse-coded and summed to calculate the total score, so that higher total scores indicated higher levels of racism-related vigilance. Internal consistency was adequate across the three waves of the present study, with Cronbach’s alphas of .80 (W1), .84 (W2), and .84 (W3).

#### Externalizing problems

Adolescents’ externalizing problems were assessed using two subscales from the 112-item Youth Self-Report (YSR; Achenbach & Rescorla, 2001), namely, Rule-breaking Behavior (14 items) and Aggressive Behavior (17 items). Adolescents were asked to report problem behaviors in the past 6 months, including the present, using a 3-point scale (0 = *Not True*; 2 = *Very True or Often True*). The YSR is a widely used measure with well-established reliability and validity. A sum score for externalizing problems was calculated using untransformed raw scores as in prior research using the YSR (Rescorla et al., 2007). Internal consistency was adequate across the three waves, with Cronbach’s alphas of .86 (W1), .86 (W2), and .85 (W3). Three items on the Anger measure were similar to three items on the Aggressive Behavior subscale (regarding temper, arguing, and being mean). To address this overlap, sum scores using a shortened version of the externalizing measure (dropping the three similar items) were also computed and used in a planned sensitivity analysis. Internal consistency of the shortened externalizing problems measure was adequate, with Cronbach’s alphas of .84 (W1), .83 (W2), and .82 (W3).

#### Internalizing problems

Adolescents’ internalizing problems were assessed using three subscales from the 112-item YSR (Achenbach & Rescorla, 2001), namely, Anxious/Depressed (13 items), Withdrawn/Depressed (8 items), and Somatic Complaints (10 items). Adolescents were asked to report problem behaviors in the past 6 months, including the present, using a 3-point scale (0 = *Not True*; 2 = *Very True or Often True*). A sum score for internalizing problems was calculated using untransformed raw scores as in prior research using the YSR (Rescorla et al., 2007). Internal consistency was adequate, with Cronbach’s alphas of .91 (W1), .93 (W2), and .91 (W3).

### Data analytic strategy

We employed latent growth curve mediation models to test the present study’s hypotheses. The predictors included direct experiences of racial/ethnic discrimination (PRaCY) or vicarious experiences of racism (VERS); mediators included anger or racism-related vigilance (HVS); and outcomes included externalizing or internalizing problems (EXT or INT). The predictors, mediators, and outcomes were all time-varying. In each model, we included one predictor, one mediator, and one outcome at a time to test the mediating mechanisms. First, a series of latent growth curve models (no change, linear, latent basis coefficient; Grimm et al., 2017; McArdle & Nesselroade, 2014) were fit to each of the time-varying variables. Global model fit indices including CFI (Bentler, 1990) and RMSEA (Browne & Cudeck, 1992) were compared to select the simplest change pattern with adequate model fit for each variable. Because we expected to observe change in each variable across the three waves, a latent intercept variable and a latent change variable were constructed to model the change trajectories for each variable. To appropriately model the temporal ordering in the mediation mechanisms, we applied different factor loadings to the latent change factors. Specifically, in the linear models, the factor loadings of the latent change factors for the input, mediator, and outcome variables were (0, .5, 1), (−0.5, 0, 0.5), and (−1, −0.5, 0) respectively. When a latent basis coefficient model was fit, for example, to an input variable, the factor loadings were (0, *, 1) with the midpoint factor loading freely estimated. With these factor loadings, the latent intercepts of the predictors, mediators, and outcomes were interpreted as initial levels at W1, midpoint levels at W2, and final levels at W3, while the latent change factors were all interpreted as the changes from W1 to W3.

**Figure 1.**
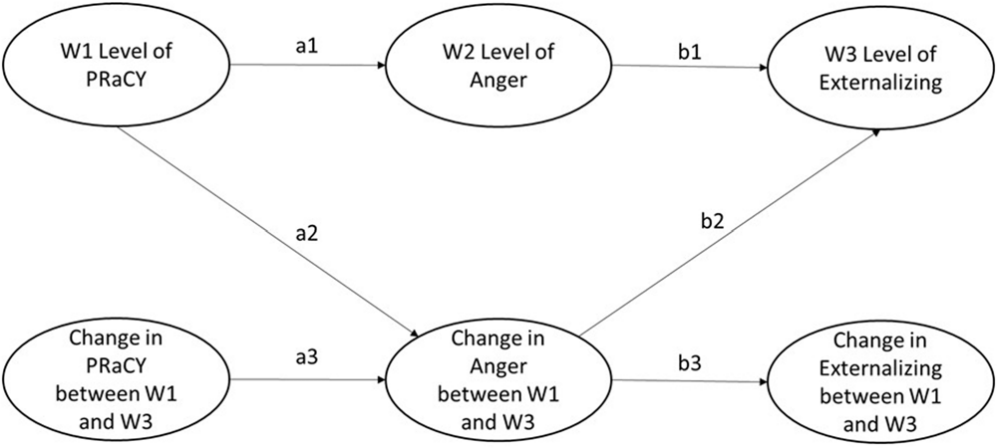
A conceptual diagram of the tested indirect effects. *Note.* Five indirect effects were tested: a1*b1, a2*b2, a2*b3, a3*b2, and a3*b3.

Latent growth curve mediation models (e.g., Cheong et al., 2003; von Soest & Hagtvet, 2011) were fitted to test the hypothesized mediation effects. To provide one example of the mediation models, we use PRaCY (direct racial/ethnic discrimination) for the predictor, ANGER (outward anger expression) for the mediator, and EXT (externalizing problems) for the outcome in the following description. In this sample model (see the conceptual model in Figure 1), the latent intercept (initial level at W1) and latent change from W1 to W3 of PRaCY predict the latent intercept (final level at W3) and latent change from W1 to W3 of EXT, measuring four direct effects (*c*1: Initial level of PRaCY → final level of EXT; *c*2: Change in PRaCY → final level of EXT; *c*3: Initial level of PRaCY → change in EXT; and *c*4: Change in PRaCY → Change in EXT).

For path *a* of the indirect effects, the latent intercept (initial level at W1) of PRaCY is modeled to predict the latent intercept (midpoint level at W2) and latent change from W1 to W3 of ANGER; the latent change from W1 to W3 of PRaCY is modeled to predict the latent change from W1 to W3 of ANGER, resulting in three *a*-path coefficients (*a1*–a3). We did not use latent change from W1 to W3 of PRaCY to predict the latent intercept (midpoint level at W2) of ANGER, as this prediction is not consistent with temporal ordering; thus, a covariance was specified to allow these two latent variables to covary. For path *b* of the indirect effects, the latent intercept (midpoint level at W2) of ANGER was modeled to predict the latent intercept (final level at W3) of EXT, and the latent change from W1 to W3 of ANGER was modeled to predict the latent intercept (final level at W3) of EXT and the latent change from W1 to W3 of EXT, resulting in three *b*-path coefficients (*b1*–*b3*). We did not use the latent intercept (midpoint level at W2) of ANGER and the latent intercept (final level at W3) of EXT to predict the latent change from W1 to W3 of EXT, as these predictions are inconsistent with temporal ordering; thus, covariances were specified to allow these two latent variables to covary with the latent change of EXT.

Two hypothesis-driven indirect effects were of primary interest:

1. initial (W1) level of PRaCY→ midpoint (W2) level of ANGER → final (W3) level of EXT (a1*b1);
2. change in PRaCY → change in ANGER → change in EXT (a3*b3).

Three other indirect effects were also estimated and tested as a function of the growth curve mediation modeling process: (a) initial level of PRaCY → change in ANGER → final level of EXT (a2*b2), (b) initial level of PRaCY → change in ANGER → change in EXT (a2*b3), and (c) change in PRaCY → change in ANGER → final level of EXT (a3*b2). We used 95% percentile bootstrap confidence intervals (CI) (Shrout & Bolger, 2002) to test the indirect effects. Youth age (grand-mean centered) and gender (Male: 0; Non-male: 1) were included as covariates for statistical controls in the models predicting the mediator latent intercept and latent change as well as the models predicting the outcome latent intercept and latent change.

Missingness in the primary variables (PRaCY, VERS, ANGER, HVS, EXT, INT) at W2 (21%–24% missingness across the variables) or W3 (23%–25% missingness) was neither significantly related to youth characteristics such as age and gender nor any of the study variables. Thus, we assumed the missing at random missingness mechanism (Rubin, 1976), and full information maximum likelihood estimation was used to estimate the models with all available data (Zhang & Wang, 2013).

Finally, planned sensitivity analyses were conducted to examine whether using the shorter measure of EXT to estimate the latent growth curve mediation models containing both ANGER and EXT would change the pattern of results. In addition, post hoc sensitivity analyses were conducted to assess whether using the unweighted version of VERS in the latent growth curve mediation models containing both VERS and INT would yield different results.

The growth curve mediation models were fit in Mplus (Muthén & Muthén, 1998–2017), and an example code is available at https://osf.io/s86f9/files/osfstorage.

## Results

### Descriptive statistics

Table 1 displays the sample sizes, sample means, and standard deviations of the primary study variables, and Table 2 shows the sample correlations. As shown in Table 1, the means of racial/ethnic discrimination increased over the three waves. In contrast, the means of other study variables (i.e., vicarious racism, anger, racism-related vigilance, and internalizing and externalizing problems) decreased over time. As expected, racial/ethnic discrimination was significantly and positively correlated with higher levels of externalizing and internalizing problems within and across waves (Table 2). Moreover, discrimination was significantly and positively correlated with anger and racism-related vigilance. Interestingly, vicarious experiences of racism were significantly and positively correlated with higher levels of *internalizing* problems within and across waves but generally *not* significantly correlated with externalizing problems. Also, vicarious experiences of racism were significantly and positively correlated with higher levels of racism-related vigilance but generally not significantly correlated with anger.

**Table 1.** Means and standard deviations of the study variables across three waves

Variables	Wave 1		Wave 2		Wave 3	
	*n*	*Mean* (*SD*)	*n*	*Mean* (*SD*)	*n*	*Mean* (*SD*)
Discrimination (PRaCY)	344	3.11 (2.22)	267	3.13 (2.39)	260	3.33 (2.42)
Vicarious racism (VERS)	325	0.91 (0.86)	249	0.77 (0.70)	247	0.69 (0.72)
Anger	344	8.01 (2.18)	267	7.97 (2.28)	261	7.72 (2.05)
Vigilance (HVS)	339	17.50 (4.97)	267	16.80 (5.26)	261	16.57 (5.34)
Externalizing problems (EXT)	344	9.71 (6.76)	266	9.18 (6.52)	261	8.70 (6.31)
Internalizing problems (INT)	344	16.07 (9.96)	266	14.90 (10.76)	261	13.16 (9.49)

**Table 2.** Correlations among the study variables across three waves

Variables	1	2	3	4	5	6	7	8	9	10	11	12	13	14	15	16	17
1. PRaCY-W1	–																
2. PRaCY-W2	.62***	–															
3. PRaCY-W3	.55***	.72***	–														
4. VERS-W1	.40***	.33***	.36***	–													
5. VERS-W2	.25***	.39***	.29***	.52***	–												
6. VERS-W3	.29***	.36***	.43***	.53***	.61***	–											
7. Anger-W1	.21***	.20**	.15*	.22***	.13*	.06	–										
8. Anger-W2	.28***	.29***	.23***	.18**	.10	.04	.57***	–									
9. Anger-W3	.20***	.23***	.22***	.08	.09	.04	.40***	.53***	–								
10. HVS-W1	.39***	.29***	.31***	.52***	.40***	.37***	.15**	.15*	.08	–							
11. HVS-W2	.44***	.45***	.40***	.37***	.50***	.38***	.16**	.17**	.11	.49***	–						
12. HVS-W3	.36***	.41***	.47***	.34***	.42***	.51***	.13*	.14*	.16**	.45***	.63***	–					
13. EXT-W1	.30***	.31***	.27***	.18**	.07	.07	.60***	.53***	.38***	.19***	.21***	.17**	–				
14. EXT-W2	.30***	.40***	.29***	.08	.04	.08	.45***	.61***	.52***	.16*	.20**	.18**	.67***	–			
15. EXT-W3	.28***	.32***	.37***	.10	.04	.09	.33***	.45***	.56***	.17**	.14*	.24***	.55***	.63***	–		
16. INT-W1	.29***	.32***	.25***	.38***	.31***	.30***	.33***	.31***	.27***	.41***	.43***	.35***	.53***	.36***	.37***	–	
17. INT-W2	.31***	.40***	.30***	.29***	.36***	.33***	.23***	.36***	.32***	.36***	.48***	.39***	.32***	.54***	.34***	.69***	–
18. INT-W3	.23***	.27***	.34***	.26***	.24***	.33***	.19**	.25***	.28***	.33***	.36***	.44***	.27***	.29***	.49***	.63***	.64***

*Note*. *: *p* < .05; **: *p* < .01; ***: *p* < .001. W1 = Wave 1; W2 = Wave 2; W3 = Wave 3. PRaCY = perception of racism in children and youth; VERS = vicarious experiences of racism; HVS = racism-related vigilance; EXT = externalizing problems; INT = internalizing problems.

### Results from growth curve mediation analyses

Comparing model fit from the three growth curve models fitted to each of the time-varying variables, the linear change model fit the data significantly better than the no change model and had an adequate fit, except for racism-related vigilance (see Table S1 in the supplement). For racism-related vigilance, the latent basis coefficient model fit the data significantly better than the linear model and had an adequate fit. Thus, we utilized the linear change function to fit the change trajectories of the time-varying variables (except for vigilance) and utilized the latent basis coefficient model for vigilance in the growth curve mediation analyses. Based on the individual growth curve models, the average change in racial/ethnic discrimination across the three waves was positive and significant (estimate = 0.139, *p* = .037). In contrast, the average changes in vicarious racism (estimate = −0.100, *p* < .001), racism-related vigilance (estimate = −0.447, *p* = .006), internalizing problems (estimate = −1.424, *p* < .001), and externalizing problems (estimate = −0.500, *p* = .006) were negative and significant. For anger, although the average change was not significant (estimate = −0.114, *p* = .091), the between-person variance in change was significant (estimate = 0.303, *p* = .039), indicating individual differences in change. Growth curve mediation analysis results are displayed in Table 3 (anger as the mediator) and Table 4 (vigilance as the mediator).

**Table 3.** Results from growth curve mediation models with anger as a mediator

Paths	Model 1 PRaCY→anger→EXT *RMSEA* = .067, *CFI* = .959		Model 2 PRaCY →anger→INT *RMSEA* = .055, *CFI* = .969		Model 3 VERS→anger→EXT *RMSEA* = .081, *CFI* = .929		Model 4 VERS→anger→INT *RMSEA* = .054, *CFI* = .968	
	Coeff	*p*-value	Coeff	*p*-value	Coeff	*p*-value	Coeff	*p*-value
**a1**: IV W1 Level → mediator W2 level	**.30**	**<.001**	**.30**	**<.001**	**.49**	**.011**	**.54**	**.007**
**a2**: IV W1 Level → mediator change	−.01	.902	−.02	.863	.00	.996	.30	.664
**a3**: IV Change → mediator change	.35	.207	.34	.123	.95	.448	1.94	.299
**b1**: Mediator W2 level → outcome W3 level	**2.53**	**<.001**	**1.47**	**.043**	**2.65**	**<.001**	**2.06**	**.001**
**b2**: Mediator change → outcome W3 level	**1.54**	**.036**	1.41	.694	**1.79**	**.017**	−8.99	.643
**b3**: Mediator change → outcome change	**2.74**	**<.001**	1.86	.755	**2.85**	**<.001**	−9.03	.400
**c1**: IV W1 Level → outcome W3 level	.47	.184	1.11	.059	.54	.820	8.44	.589
**c2**: IV Change → outcome W3 level	.88	.428	1.50	.530	.11	.988	26.23	.652
**c3**: IV W1 Level → outcome change	−.09	.727	−.37	.476	.51	.779	3.74	.765
**c4**: IV Change → outcome change	.27	.567	1.48	.716	−.21	.972	25.04	.528

*Note*. W1 = Wave 1; W2 = Wave 2; W3 = Wave 3. IV = independent variable. PRaCY = perception of racism in children and youth; VERS = vicarious experiences of racism; EXT = externalizing problems; INT = internalizing problems. Coeff = path coefficient estimate. The bolded paths indicate statistical significance at the .05 level (i.e., *p* < .05).

**Table 4.** Results from growth curve mediation models with vigilance as a mediator

Paths	Model 5 PRaCY→vigilance→EXT *RMSEA* = .054, *CFI* = .969		Model 6 PRaCY→vigilance→INT *RMSEA* = .056, *CFI* = .970		Model 7 VERS→vigilance→EXT *RMSEA* = .040, *CFI* = .982		Model 8 VERS→vigilance→INT *RMSEA* = .059, *CFI* = .967	
	Coeff	*p*-value	Coeff	*p*-value	Coeff	*p*-value	Coeff	*p*-value
**a1**: IV W1 Level → mediator W2 level	**1.29**	**<.001**	**1.28**	**<.001**	**3.24**	**<.001**	**3.25**	**<.001**
**a2**: IV W1 Level → mediator change	.13	.642	.10	.714	**2.43**	**.009**	**2.48**	**.013**
**a3**: IV Change → mediator change	**1.83**	**.031**	**1.98**	**.004**	**7.28**	**<.001**	**7.58**	**<.001**
**b1**: Mediator W2 level → outcome W3 level	−.27	.817	.80	.633	2.28	.178	2.55	.257
**b2**: Mediator change → outcome W3 level	1.12	.910	1.49	.918	−8.01	.524	−6.44	.732
**b3**: Mediator change → outcome change	.10	.921	.13	.936	−.04	.978	−.62	.773
**c1**: IV W1 Level → outcome W3 level	1.46	.645	.32	.936	8.97	.758	8.59	.856
**c2**: IV Change → outcome W3 level	−.71	.967	−1.12	.970	49.89	.574	44.95	.751
**c3**: IV W1 Level → outcome change	−.09	.834	−.47	.435	.42	.922	2.07	.744
**c4**: IV Change → outcome change	.90	.615	2.08	.551	2.14	.845	10.11	.568

*Note*. W1 = Wave 1; W2 = Wave 2; W3 = Wave 3. IV = Independent Variable; PRaCY = perception of racism in children and youth; VERS = vicarious experiences of racism; EXT = externalizing problems; INT = internalizing problems. Coeff = path coefficient estimate. The bolded paths indicate statistical significance at the .05 level (i.e., *p* < .05).

#### Anger as mediator

Results indicated that higher initial levels of direct experiences of racial/ethnic discrimination or vicarious experiences of racism at Wave 1 were associated with higher levels of anger at Wave 2, which in turn were associated with higher levels of externalizing or internalizing problems at Wave 3. Specifically, anger at W2 significantly mediated the relations between: (a) racial/ethnic discrimination at W1 and final level of externalizing problems at W3 (*a1* = 0.303, *p* < .001; *b1* = 2.534, *p* < .001; 95% CI of a1*b1: [0.375, 1.339]); (b) racial/ethnic discrimination at W1 and final level of internalizing problems at W3 (*a1* = 0.299, *p* < .001; *b1* = 1.470, *p* = 0.043; 95% CI of a1*b1: [0.051, 1.049]); (c) vicarious racism at W1 and final level of externalizing problems at W3 (*a1* = 0.489, *p* = .011; *b1* = 2.646, *p* < .001; 95% CI of a1*b1: [0.415, 2.778]); and (d) vicarious racism in W1 and final level of internalizing problems at W3 (*a1* = 0.535, *p* = .007; *b1* = 2.065, *p* = 0.001; 95% CI of a1*b1: [0.342, 2.548]). The *b3* paths from change in anger to change in externalizing problems were statistically significant (*p* < 0.001; see Models 1 and 3 in Table 3), indicating that less decline in anger was associated with less decline in externalizing problems. The associated indirect effects (a2*b3 and a3*b3; see Figure 1) were not significant because *a2* and *a3* were not significant. The *b2* paths from change in anger to the final level of externalizing problems were statistically significant (*p* = 0.036 and 0.017; see Models 1 and 3 in Table 3), indicating that less decline in anger was associated with a higher final level in externalizing problems. The associated indirect effects (a2*b2 and a3*b2; see Figure 1) were not statistically significant due to the nonsignificant *a2* and *a3*.

Table S2 shows the results from the planned sensitivity analyses using the shortened version of the externalizing problems measure. The pattern of significant indirect effects remained the same as those reported above. Specifically, the level of anger at W2 significantly mediated the relations between: (a) racial/ethnic discrimination at W1 and the final level of externalizing problems (the shorter version) at W3 (*a1* = 0.304, *p* < .001; *b1* = 1.926, *p* < .001; 95% CI of a1*b1: [0.278, 1.047]); and (b) vicarious racism at W1 and the final level of externalizing problems (the shorter version) at W3 (*a1* = 0.491, *p* = .010; *b1* = 2.040, *p* < .001; 95% CI of a1*b1: [0.320, 2.252]). The only difference was that the *b2* path became non-significant with the shorter Externalizing Problems measure; that is, change in anger was no longer significantly associated with the final level of externalizing problems (*p* = 0.065 and 0.108; see Table S2).

Results from the post hoc sensitivity analyses (Table S3, Model 4: VERS → Anger → INT) indicated that the pattern of significant indirect effects remained the same when the unweighted version of the Vicarious Experiences of Racism Scale (VERS) was used. Specifically, the level of anger at W2 significantly mediated the associations between VERS (unweighted) at W1 and the final levels of internalizing problems at W3 (*a1* = 0.571, *p* < .001; *b1* = 1.943, *p* < .001; 95% CI of a1*b1: [0.421, 1.799]). However, the *b2* and *b3* paths became significant, indicating that less decline in anger became significantly and positively associated with both a higher final level of, and less decline in, internalizing problems (*p* = 0.029 and 0.003, respectively), after controlling for the initial level and change of VERS (unweighted).

#### Racism-related vigilance as mediator

The four *a1* paths from direct experiences of racial/ethnic discrimination as well as vicarious experiences of racism in W1 to racism-related vigilance in W2 were all statistically significant (*p* < 0.001; see Models 5–8 in Table 4), indicating that a higher level of direct racial/ethnic discrimination and a higher level of vicarious racism at W1 were associated with higher levels of racism-related vigilance in W2. The two *a2* paths from vicarious racism in W1 to change in racism-related vigilance over time were both statistically significant (see Models 7–8 in Table 4), indicating that a higher level of vicarious racism in W1 was associated with less decline in racism-related vigilance over time. The four *a3* paths from change in direct racial/ethnic discrimination or change in vicarious racism over time to change in racism-related vigilance over time were all statistically significant (see Models 5–8 in Table 4, IV change → mediator change), indicating that greater increases in direct racial/ethnic discrimination or greater increases in vicarious racism over time were associated with less decline in racism-related vigilance over time. However, none of the indirect effects were statistically significant because none of the *b* paths from racism-related vigilance to internalizing or externalizing problems were statistically significant.

The post hoc sensitivity analyses using the unweighted version of VERS (Table S3, Model 8: VERS → Vigilance → INT) produced different results. Specifically, the level of vigilance at W2 significantly mediated the association between VERS (unweighted) at W1 and the final level of internalizing problems at W3 (*a1* = 2.702, *p* < .001; *b1* = 1.457, *p* < .001; 95% CI of a1*b1: [2.142, 5.734]). This significant indirect effect was due to the *b1* path becoming significant after controlling for the initial level and change in VERS (unweighted). In addition, the *a2* and *a3* paths linking the initial level and change in VERS (unweighted) to change in vigilance became nonsignificant (*p*s > .376). However, the *b3* path (the effect of change in vigilance on change in internalizing problems) became significant (*b3* = .887, *p* = .012), indicating that less decline in vigilance was associated with less decline in internalizing problems after controlling for the initial level and change in VERS (unweighted).

## Discussion

The purpose of the present longitudinal study was to identify mediating mechanisms that help explain the association between racism and mental health problems among Mexican-origin adolescents. Specifically, we tested two transdiagnostic mediators – anger expression and racism-related vigilance – of the association between direct and vicarious racism and internalizing and externalizing problems. This study is novel because it represents the first longitudinal test of these transdiagnostic mediating mechanisms in a sample of Latinx adolescents; it is also innovative in the longitudinal design which permitted tests of both mediating mechanisms and change processes over time.

As hypothesized, results indicated that anger (at Wave 2) was a significant and consistent mediator in the association between both direct and vicarious racial/ethnic discrimination (at Wave 1) and internalizing and externalizing problems (at Wave 3). Higher initial levels of direct or vicarious racism were associated with higher midpoint levels of anger, which in turn were associated with higher final levels of internalizing and externalizing problems, respectively. Moreover, changes in anger were also associated with changes in externalizing problems, such that less decline in anger over time was associated with less decline in externalizing symptoms over time. These significant change-related processes were consistently observed, even when the overlapping items between the anger and externalizing symptoms measures were removed. Theoretically, these findings are in line with Harrell’s (2000) model which posits that racism can manifest in different forms (including direct and vicarious experiences) and adversely affect various psychological and behavioral outcomes. Harrell (2000) highlighted the mediating role of affective responses such as anger, and the current findings provide support for this component of the model. The Mexican-origin adolescents in the present sample responded to both direct and vicarious racism with anger, the emotion most frequently evoked in the face of injustice (Mikula et al., 1998). In turn, anger was associated with both internalizing and externalizing problems. According to Harrell (2000), emotional responses (such as anger) to various forms of racism can lead to different types of mental health outcomes depending on the choice of different coping strategies, the nature of the surrounding environment (e.g., hostile or receptive), and the availability of support networks or resources.

Empirically, these results both confirm and extend results from prior research. These results are congruent with past research in that outward anger expression has been found to be a significant mediator of the discrimination – mental health link in two prior studies (Park et al., 2017, 2024). The present study extends the existing knowledge base by expanding the scope of the tested variables (i.e., testing direct and vicarious forms of discrimination; assessing internalizing and externalizing problems), extending the time scale, and using growth curve mediation modeling which permitted a more in-depth examination of macro-time change processes. Although anger is an appropriate response to injustice such as racism, there is a cost in terms of adolescent mental health as this combined research indicates that anger is also associated with more distress in the short-term (Park et al., 2024) as well as more internalizing and externalizing problems over the long term. In sum, the present findings show that anger is a consistent mediating mechanism that links both direct and vicarious discrimination to both internalizing and externalizing problems, respectively, in this sample of Mexican-origin adolescents.

Contrary to our hypotheses, the present results revealed that racism-related vigilance was *not* a significant mediator of the association between either direct or vicarious experiences of discrimination and racism and either internalizing or externalizing problems in this sample of Mexican-origin adolescents. This null finding adds to mixed results from prior research (e.g., Mikrut et al., 2022; Pichardo et al., 2021). At the same time, results from a post hoc sensitivity analysis using the unweighted version of the vicarious racism measure revealed one significant indirect effect. Specifically, racism-related vigilance significantly mediated the effect of vicarious racism on internalizing problems (Table S3). Higher levels of vicarious racism experienced at W1 were significantly associated with higher levels of racism-related vigilance at W2, which in turn, were significantly associated with higher levels of internalizing problems at W3 in this sample.

One possible explanation for this significant mediation effect is that vicarious (versus direct) racism is more “free-floating” as it is not targeting the adolescent themselves but involves people whom the adolescent may know either proximally and intimately (e.g., family members) or distally as representatives from the same ethnic group (e.g., athletes, celebrities). Consequently, Mexican-origin adolescents may feel less control and attempt to increase monitoring efforts expressed through racism-related vigilance to protect themselves. In turn, exercising sustained levels of vigilance may tax adolescents’ coping resources, reinforcing the same processes that later develop into internalizing symptoms (e.g., rumination, worry; Woody et al., 2022). Given the different results obtained from the two versions of the vicarious racism measure (with and without the impact items), more research is necessary to replicate these findings. Since the unweighted vicarious racism measure removes potential bias, the significant mediation effect of racism-related vigilance in the vicarious racism – internalizing problems link merits attention.

These post hoc sensitivity results are also consistent with statistical theory. In mediation models, the *b* paths represent the effects of mediators on outcomes after controlling for input variables. When the original weighted version of the Vicarious Experiences of Racism Scale (VERS) was used, part of the variance in the final level of internalizing problems that vigilance at W2 could explain is already accounted for by the initial level of VERS (weighted) due to the “impact” items. As a result, the *b* path can be underestimated or nonsignificant, even if vigilance genuinely affects internalizing problems.

Further, two sets of statistically significant change processes emerged. First, higher initial levels of vicarious (but not direct) racism at Wave 1 were significantly associated with less decline in racism-related vigilance over time. Second, *changes* in direct and vicarious discrimination experiences over time were significantly associated with *changes* in racism-related vigilance over time. More specifically, *increases* over time in direct and vicarious racism were significantly associated with *less decline* in racism-related vigilance over time. This is an important and novel contribution to the literature, moving beyond prior research (e.g., Park et al., 2017) because these associations are capturing *change* processes (not merely linear trends) over time. One reason for this significant association may be that when adolescents experience increases in direct and vicarious racism, they may recognize the need to maintain steady levels of vigilance (e.g., monitoring their behaviors and their environment) to avoid any feared negative consequences.

Results from the post hoc sensitivity analyses indicated additional significant change processes. Less decline in racism-related vigilance or anger was significantly associated with less decline in internalizing problems when the independent variable was (unweighted) vicarious racism (see Table S3). This finding provides empirical evidence that changes in either mediating mechanism (anger or racism-related vigilance) influences changes in internalizing problems, in line with theoretical expectations (e.g., Brondolo et al., 2018; Harrell, 2000). These paired change processes have important clinical implications because targeting either racism-related vigilance or anger in intervention efforts may be helpful in reducing internalizing symptoms.

As with the mediation results, the results regarding change processes varied depending on whether or not the unweighted version of the VERS was utilized. Thus, it will be important in future research to replicate these results and select the most appropriate version of the VERS depending on the mental health outcomes of interest. If the mental health outcome involves distress or internalizing symptoms, we recommend the use of the unweighted version of the VERS to avoid potential bias.

Taken together, these results underscore the toll that racism takes on the mental health of Mexican-origin adolescents, given the costs associated with sustained levels of racism-related vigilance. Integrating findings from both the planned and post hoc sensitivity analyses, racism-related vigilance emerged as a significant mediating mechanism in the link between vicarious racism and internalizing problems. These findings highlight the value of considering both vicarious and direct racism, the importance of disentangling frequency versus impact of racism especially in detecting associations with internalizing symptoms, and the advantage of a longitudinal design in capturing change over time.

### Study strengths and limitations

The present study makes several contributions to the current literature on mediating mechanisms that explain the link between racism and adolescent mental health. First, we tested two transdiagnostic mediating mechanisms that have explanatory power across a variety of potential mental health outcomes and the development of psychopathology. The principle of multifinality (Cicchetti & Rogosch, 1996) is in view here as exposure to racial/ethnic discrimination can lead to multiple kinds of outcomes that affect adolescent mental health. Second, and relatedly, the present study captured both externalizing and internalizing problems as mental health outcomes that are typically precursors to more severe forms of psychopathology (van der Ende et al., 2020). Third, the present study assessed both direct experiences of racial/ethnic discrimination and vicarious exposure to racism. This is important as both first-hand and vicarious or “second-hand” experiences of racism and discrimination can have detrimental effects on adolescent mental health (Heard-Garris et al., 2018), as borne out by the present study’s findings. Next, the use of growth curve mediation analyses afforded the opportunity to not only test the hypothesized mediation models but also tap into related change processes associated with these mediation pathways. Finally, the current sample of Mexican-origin adolescents living in the Midwestern United States represents an under-researched population, both developmentally and geographically. Thus, the present study provides a window into a critical developmental transition period for these youths who live in a relatively new migration destination area. The state of Indiana has a more exclusionary immigration policy climate which has been associated with poor mental health (Hatzenbuehler, 2017); the Midwestern United States may also have less infrastructure and support for Mexican immigrant families (Espinola et al., 2019), compared to metropolitan areas such as New York City, Miami, Los Angeles or areas with more concentrated populations of Mexican-origin immigrant families such as the Southwestern United States.

It is important to interpret the present study’s results in light of its limitations. First, we acknowledge that, despite the benefits of pinpointing potential targets for intervention and prevention purposes, one pitfall of focusing on intrapersonal explanatory mechanisms (e.g., anger, racism-related vigilance) is the danger of assuming a victim-blame mentality, which we do not endorse. Instead, we advocate for a multi-level, multi-pronged approach to eradicating racism and racial/ethnic discrimination both at the structural and societal levels as well as at the interpersonal and individual levels. Second, as the sampling strategy for the larger parent study (Alegría et al., 2024) focused on a defined geographic area, the generalizability of the results may be restricted. However, the present study’s findings contribute to the paucity of longitudinal research on the effects of racism and discrimination over time using more rigorous analytical methods, such as growth curve mediation analyses, among Mexican-origin youth who live in relatively new migration areas. Despite the use of advanced analytic methods, the observational design of the present study restricts the degree to which causal inferences can be made (Liu & Wang, 2024); future research may focus on gathering additional time points to enable more precise conclusions about the sequence of events and processes in the discrimination–mental health pathway. Shared method variance is another possible limitation given that the study relied upon single informant measures. However, at least two factors mitigate this concern in this sample, including: (a) several constructs (e.g., experiences of discrimination, internalizing symptoms) are more accurately assessed using self-report; and, (b) distinct patterns emerged in the model (e.g., not every youth-reported variable was related to every other variable). In future research, the use of multiple reporters can help address this issue of shared method variance. Finally, for some of the study variables (e.g., mental health outcomes), the means appeared to decrease over the three waves. Still, we also observed increasing discrimination over the three waves. History effects may explain these trends. The COVID-19 pandemic overlapped with some of the present study’s data collection period (April 2021 through October 2024); thus, decreasing internalizing and externalizing symptoms may be due to data collection beginning at a time of heightened distress (i.e., the COVID-19 pandemic) through a period of recovery. This time period also coincided with major historical events (e.g., the murder of George Floyd), which highlighted and exacerbated experiences of racism.

### Implications for future theory, research, and practice

The present study’s findings have significant implications for future theory-building, research, and clinical practice. The study hypotheses were generated by integrating theoretical models drawn from the social cognition (Brondolo et al., 2018) and stress and coping literatures (Harrell, 2000); the focus on transdiagnostic mediators also bridges to the developmental psychopathology literature (Beauchaine & Cicchetti, 2019; Roach et al., 2023; Woody et al., 2022). Given the multiple levels at which racism can operate and their wide-ranging effects on adolescent mental health, we recommend greater integration across these diverse yet related literatures for future theory-building. Integrative theory-building may then yield greater insights into how racism and discrimination get “under the skin” to adversely affect the mental health of racially and ethnically minoritized populations.

The present study findings offer insights and avenues for future research. First, methodologically, the distinction between the weighted versus unweighted versions of the VERS highlights a broader assessment issue in the field – that is, the tension between assessing exposure/frequency versus impact of racism and discrimination. In the present study, both exposure to vicarious racism and its impact were addressed, and results revealed that operationalization matters for associations with internalizing symptoms. These findings can aid future researchers in weighing their own instrumentation and analytical decisions toward the goal of elucidating consequences of racism on mental health. Second, other components of emotion (dys-)regulation more broadly (e.g., rumination) or other forms of anger coping more specifically (e.g., anger suppression) should be examined as potential mediators of the association between racism and adolescent mental health. Third, future research should consider including both mediators (i.e., racism-related vigilance and anger) in the same model. As one anonymous reviewer proposed, a hypothetical pathway from racial/ethnic discrimination to racism-related vigilance to anger to externalizing problems could be tested. Fourth, both internalizing and externalizing problems should be tested in the same model, given strong interest in unique versus co-occurring effects and processes in the field of developmental psychopathology. Although we attempted this as a post hoc analysis, the models failed to converge. Next, a reverse association is possible between race-related stress and trauma and ensuing adjustment problems (Roach et al., 2023). Specifically, not only is racial/ethnic discrimination associated with emotion dysregulation and in turn, mental health problems such as externalizing behaviors, but also emotion dysregulation may be associated with perceiving more race-related stress and trauma and increased vulnerability to poor mental health outcomes. Thus, future studies may consider testing bidirectional or reciprocal effects between these variables. Future research should also examine more time points through intensive longitudinal designs to draw more precise conclusions regarding the sequence of events and directionality. Finally, future research should continue to investigate the change processes involved in the discrimination – mental health link over time.

The present findings have implications for clinical practice. The results clearly indicated that regardless of whether an adolescent’s exposure to racism is direct or vicarious, anger is aroused, which in turn, is robustly associated with both internalizing and externalizing problems. Clinical practitioners should be aware of how potent and detrimental any kind of racially charged mistreatment can be, whether experienced first-hand or indirectly. Anger is a normal response to unfair treatment and injustice (Mikula et al., 1998). Thus, before attempting to reduce anger expression through intervention efforts among Mexican-origin adolescents who are coping with racism, it is important that clinicians assess the adolescent’s ecological context to discern what would be most adaptive and appropriate (Roach et al., 2023). Outward anger expression, especially at high and sustained levels over time, can take a toll on adolescent mental health. Therefore, clinicians may consider how to best help Latinx adolescents coping with racism to express their anger in a healthy manner in safe spaces and with safe people, to redirect their anger toward mobilization/advocacy or other strategies that are empowering to the individual (Harrell, 2000). Clinicians should also seek to develop their competence and confidence in discussing racism-related events with youth in ways that support positive youth development (Galán et al., 2022). Although the empirical literature on the effectiveness of emotion regulation interventions is nascent at best, a recent meta-analysis of 21 randomized control trials among youths showed some evidence (small to moderate effect sizes) that emotion regulation can be improved following intervention (Moltrecht et al., 2021). Caution should be exercised, however, before implementing these emotion regulation interventions in a “one size fits all” manner, given that there is a critical need for more research to understand “what works for who and when” (Moltrecht et al., 2021, p. 844).

The findings regarding racism-related vigilance have different clinical implications. Sustained levels of racism-related vigilance can be harmful to adolescents, given the depletion of cognitive and emotional resources over time, increased risk for depression, and reduced functioning in myriad domains including school, sleep, social interactions, and chronic stress (Woody et al., 2022). The finding that more experiences of either direct or vicarious racism at Wave 1 were associated with more racism-related vigilance at Wave 2 and that increases in both types of racism were associated with less declines in vigilance reveal how closely linked racism and vigilance are over time. Vicarious racism seems especially pernicious in evoking vigilance because both higher levels of vicarious racism (at Wave 1) and changes in vicarious racism were significantly associated with higher levels of vigilance (at Wave 2) and less decline in vigilance over time. High levels of vigilance or less declines in vigilance indicate that adolescents are anxiously monitoring their environment and their own behaviors as a result of direct and indirect exposures to racism. Clinicians who serve Latinx adolescents should be aware that even vicarious exposure to discrimination experiences can elicit these vigilant behaviors. One way to disrupt the association between racism and vigilance may be through mindfulness-based therapeutic techniques that reduce anxiety arousal in the wake of exposure to direct or vicarious racism (Woody et al., 2022). Two recent scoping reviews addressing individual-level interventions for reducing racial bias & discrimination (Guo et al., 2023) and interventions addressing systemic racism (Matos et al., 2024) also offer some potential avenues for intervention efforts. One noteworthy example is the Engaging, Managing, and Bonding through Race (EMBRace) intervention (Anderson et al., 2019); it is a theoretically grounded, empirically supported, culturally congruent family-based racial socialization intervention that may help reduce racism-related vigilance. Developed for Black parents and adolescents, it is a promising model that can guide future intervention research. Finally, policymakers should also advocate for ways to lower vicarious exposure to racial/ethnic discrimination at a systemic level, in a manner similar to efforts toward reducing the dangerous physical health effects of exposure to second-hand smoke.

In conclusion, results from the present three-wave longitudinal study of Mexican-origin adolescents indicated that anger was a significant mediator in the racism – mental health link over time, for both direct and vicarious exposure to racism and both internalizing and externalizing problems. Racism-related vigilance was a significant mediator of the link between vicarious racism and internalizing symptoms only. The study makes important strides toward a more integrated theoretical understanding of how exposure to racism can have adverse effects on adolescent mental health and provides essential insights for clinicians and policymakers considering how to help ethnically minoritized youths cope with racism in a new migration area.

## Supporting information

10.1017/S0954579426101163.sm001 Park et al. supplementary material Park et al. supplementary material

## Data Availability

Availability of Data: The data necessary to reproduce the analyses presented here are not publicly accessible for the safety of the study population. The authors are not able to release data used in the current manuscript given the sensitivity of the data and their agreements with the Institutional Review Boards of the participating institutions. Availability of Code: The analytic code necessary to reproduce the analyses presented in this paper is publicly accessible and may be found on the Open Science Framework website here: https://osf.io/s86f9/files/osfstorage. Availability of Methods/Material: The materials necessary to attempt to replicate the findings presented here are not publicly accessible; however, materials are available upon request from the first or last author.

## Supplementary material

The supplementary material for this article can be found at https://doi.org/10.1017/S0954579426101163.

## Preregistration

The present study’s hypotheses and analytic plan were not pre-registered. However, the current study’s mediation hypotheses and growth curve analyses were proposed a priori (i.e., in our NIH grant application). The overall objective of the parent study, description of the parent study aims as well as study design, eligibility criteria, and key measures were posted on clinicaltrials.gov at the following link: https://clinicaltrials.gov/study/NCT04875208.
