# Supplementary material for: Mediating mechanisms in the discrimination – Mental health link among Mexican-origin adolescents: A latent growth curve mediation analysis
Source: Dev Psychopathol. 2026 Feb 3:1–14. Online ahead of print. doi: 10.1017/S0954579426101163 (PMC12931430; doi:10.1017/S0954579426101163)
Supplement: Park et al. supplementary material [file S0954579426101163sup001.docx]

**Table S1**

*Growth curve model fit indices*

| Variables | No change model | | | Linear change model | | | Latent basis coefficient model | | |
| --- | --- | --- | --- | --- | --- | --- | --- | --- | --- |
|  | ${}^{2}$ | *RMSEA* | *CFI* | ${}^{2}$ | *RMSEA* | *CFI* | ${}^{2}$ | *RMSEA* | *CFI* |
| Discrimination (PRaCY) | **25.11** | .096 | .938 | 6.68 | .060 | .988 | 3.82 | .051 | .994 |
| Vicarious racism (VERS) | **49.61** | .146 | .785 | **18.26** | .122 | .925 | 1.45 | .000 | 1.00 |
| Anger | **15.13** | .067 | .951 | 6.79 | .061 | .979 | 5.54 | .072 | .981 |
| Vigilance (HVS) | **22.03** | .088 | .917 | 6.74 | .060 | .981 | .16 | .000 | 1.00 |
| Externalizing problems (EXT) | **19.07** | .080 | .956 | 1.04 | .000 | 1.00 | na | na | na |
| Internalizing problems (INT) | **42.16** | .132 | .892 | 9.29 | .062 | .984 | na | na | na |

*Note*. Bolded chi-squared test statistics indicate a statistically significant result (i.e., *p* < .05), meaning that this model fits significantly worse than the saturated (perfectly fitting) model. na: not available due to nonconvergence.

**Table S2**

*Sensitivity analysis results with the shorter version of the externalizing problem measure*

| Paths | Model 1  PRaCY→Anger→EXT  *RMSEA* = .059, *CFI* = .966 | | Model 3  VERS→Anger→EXT  *RMSEA* = .076, *CFI* = .932 | |
| --- | --- | --- | --- | --- |
|  | *Coeff* | *p-value* | *Coeff* | *p-value* |
| **a1**: IV W1 Level → Mediator W2 Level | **.30** | **<.001** | **.49** | **.010** |
| **a2**: IV W1 Level → Mediator Change | -.01 | .912 | .00 | .990 |
| **a3**: IV Change → Mediator Change | .35 | .294 | .96 | .471 |
| **b1**: Mediator W2 Level → Outcome W3 Level | **1.93** | **<.001** | **2.04** | **<.001** |
| **b2**: Mediator Change → Outcome W3 Level | 1.40 | .065 | 1.67 | .108 |
| **b3**: Mediator Change → Outcome Change | **2.30** | **<.001** | **2.41** | **.003** |
| **c1**: IV W1 Level → Outcome W3 Level | .48 | .130 | .51 | .843 |
| **c2**: IV Change →Outcome W3 Level | .76 | .461 | -.14 | .987 |
| **c3**: IV W1 Level → Outcome Change | -.06 | .783 | .44 | .836 |
| **c4**: IV Change → Outcome Change | .27 | .822 | -.16 | .981 |

*Note*. W1 = Wave 1; W2 = Wave 2; W3 = Wave 3. IV = Independent Variable. PRaCY = Perception of Racism in Children and Youth; VERS = Vicarious Experiences of Racism; EXT = Externalizing problems. Coeff = path coefficient estimate. The bolded paths indicate statistical significance at the .05 level (i.e., *p* < .05).

**Table S3**

*Sensitivity analysis results with the unweighted version of the vicarious racism measure*

| Paths | Model 4  VERS→Anger→INT  *RMSEA* = .046, *CFI* = .976 | | Model 8  VERS→Vigilance→INT  *RMSEA* = .047, *CFI* = .978 | |
| --- | --- | --- | --- | --- |
|  | *Coeff* | *p-value* | *Coeff* | *p-value* |
| **a1**: IV W1 Level → Mediator W2 Level | **.57** | **< .001** | **2.70** | **< .001** |
| **a2**: IV W1 Level → Mediator Change | -.23 | .392 | -.04 | .964 |
| **a3**: IV Change → Mediator Change | -1.34 | .245 | -3.70 | .380 |
| **b1**: Mediator W2 Level → Outcome W3 Level | **1.94** | **< .001** | **1.46** | **< .001** |
| **b2**: Mediator Change → Outcome W3 Level | **2.18** | **.029** | .46 | .191 |
| **b3**: Mediator Change → Outcome Change | **2.67** | **.003** | **.89** | **.012** |
| **c1**: IV W1 Level → Outcome W3 Level | 1.51 | .078 | -1.95 | .064 |
| **c2**: IV Change →Outcome W3 Level | .50 | .813 | -.68 | .718 |
| **c3**: IV W1 Level → Outcome Change | -.23 | .788 | -1.02 | .214 |
| **c4**: IV Change → Outcome Change | -.32 | .899 | -1.07 | .665 |

*Note*. W1 = Wave 1; W2 = Wave 2; W3 = Wave 3. IV = Independent Variable. VERS = Vicarious Experiences of Racism; EXT = Externalizing problems; INT = Internalizing problems. Coeff = path coefficient estimate. The bolded paths indicate statistical significance at the .05 level (i.e., *p* < .05).
